# Mucosal BCG delivery provides a spectrum of protection from different *Mycobacterium tuberculosis* strains across susceptible and resistant mouse backgrounds

**DOI:** 10.3389/ftubr.2024.1417939

**Published:** 2024-10-15

**Authors:** Sasha E. Larsen, Brittany D. Williams, Tiffany Pecor, Valerie A. Reese, Zhiyi Zhu, Dana E. Miller, Brendan K. Podell, Susan L. Baldwin, Rhea N. Coler

**Affiliations:** ^1^Seattle Children's Research Institute, Center for Global Infectious Disease Research, Seattle Children's, Seattle, WA, United States; ^2^Department of Global Health, University of Washington, Seattle, WA, United States; ^3^Department of Microbiology, Immunology and Pathology, Colorado State University, Fort Collins, CO, United States; ^4^Department of Pediatrics, University of Washington School of Medicine, Seattle, WA, United States

**Keywords:** BCG, *Mycobacterium tuberculosis*, tuberculosis, lineages, mouse strain, vaccination

## Abstract

*Mycobacterium bovis* Bacille Calmette-Guérin (BCG) is the sole globally licensed vaccine against tuberculosis despite its relatively moderate protection of acute disease through adolescence. We hypothesize that vaccine efficacy from a mucosal BCG vaccination will be directly influenced by *Mycobacterium tuberculosis* (*M.tb*) strain and mouse background. Here we investigated the effectiveness of mucosal BCG vaccination via the intranasal route, in resistant and susceptible mouse strains, to protect against laboratory strain H37Rv and clinical strain HN878 *M.tb* aerosol challenge. We evaluated both pulmonary and disseminated CFU at 4-weeks post-infection in addition to survival endpoints in C57BL/6, SWR, and C3HeB/FeJ mice. Antigen specific T cell responses in the lung post-infection were also evaluated. We observed that in each case intranasal BCG afforded a significant reduction in pulmonary CFU at 4-weeks post-infection compared to matched untreated controls. However, only susceptible mouse strains, SWR and C3HeB/FeJ, demonstrated similarly robust control from bacterial dissemination when CFU in the spleen was evaluated at the same timepoint. In the case of both *M.tb* H37Rv and *M.tb* HN878 challenge, intranasal BCG significantly improved survival of each mouse cohort compared to unvaccinated controls. Together these data suggest that there is still much to be learned from the century old vaccine, BCG, and how it drives protection.

## Introduction

*Mycobacterium bovis* Bacille Calmette-Guérin (BCG) remains the sole licensed vaccine against *Mycobacterium tuberculosis* (*M.tb*), despite noted waning protection from disease (1–3). This is in part due to mixed efficacy reporting that may be due to factors like which BCG strains are used for vaccination (4, 5), composition of host genetics or prior exposures to other environmental mycobacteria (6–8) or other variables related to *M.tb* virulence (9, 10). Studies of outbreaks with known exposures to *M.tb* suggest BCG vaccination can result in reduced risk of test conversion in a gamma interferon release assay, T-SPOT^^®^^.TB-test, including in a school setting in the UK (11). Therefore, BCG is still deployed in nations with endemic *M.tb*. BCG alone has been unable to halt the population-level progression of tuberculosis disease (TB) that ravages low and middle income countries disproportionately (12, 13). Novel TB vaccine candidates have stalled through clinical and preclinical pipelines due to a lack of robust and tangible immune correlates for stage-gating advancement (14–16). Preceding novel vaccine candidates, the research community needs to leverage novel vaccine systems, routes of delivery and host genetics to identify the requirements for durable protection from TB disease.

While BCG does not provide durable immunity from TB disease or prevent infection, there are still ample ways in which immunologists and vaccine developers can leverage this century old vaccine to winnow down the most critical features of protective immunity, including in preclinical models. After the Lübeck disaster (17, 18), less effort was focused on mucosal BCG delivery as a mechanism for correlates of protection discovery. However, over the last two decades a trove of data has been shared in this regard. For example, Perdomo and colleagues demonstrated that lung resident memory T cells were induced by intratracheal immunization with BCG in a C57BL/6 mouse model (19). Intranasally (i.n.) delivered BCG is a standard control group for mycobacterial growth inhibition assays (MGIA) using *ex vivo* mouse samples for surrogate efficacy endpoints (20–22) and has been shown to protect BALB/c mice partially via accelerating IFNγ production at the pulmonary site (23). Others have shown that protection induced by mucosal BCG vaccination in susceptible DBA/2 mice is partially IL-17-dependent (24), and in other studies specifically drives critical cell homing to the lungs [reviewed here (25)]. The purpose of this study is to expand the understanding of the protective effect of i.n. vaccination with BCG against laboratory and clinical strains of *M.tb* across three different mouse strains with varying susceptibility to infection, C57BL/6, SWR, and C3HeB/FeJ.

Recent advancements in interrogating host genetics include the development and sharing of collaborative cross (CC) mice, which are genetically diverse but tractable, enabling identification of loci that correlate with protective responses to infection (26, 27). In CC and conventional mouse strains, a spectrum of sensitivity to *M.tb* infection is observed. In these studies, C57BL/6 mice are relatively resistant (low bacterial burden in tissues) to an intravenous (i.v.) challenge with *M.tb* H37Rv vs. other strains (28). However, when examining the protection afforded by subcutaneous (s.c.) BCG vaccination, male C57BL/6 were protected (>0.5 log_10_ CFU reduction) in both the lung and spleen 4 and 14 weeks post aerosol challenge with *M.tb* H37Rv [Figure 3 here (29)]. Despite their relative resistance to *M.tb*, C57BL/6 have been extensively used as a base challenge mouse strain when evaluating different *M.tb* strains, TB vaccines and immunity (30–32). Swiss inbred SWR mice, conversely, are well-known for being particularly sensitive to *M.tb* challenge which seems partially linked to defects in complement C5, inclusions in alveolar macrophages and worse disease progression due to accumulation of less functional effector T cells (30, 33–35). The C3HeB/FeJ mouse model is another canonically leveraged mouse strain given that they more faithfully recapitulate specific types of human-like necrotic TB lesions (36–39). C3HeB/FeJ mice (also referred to as Kramnik mice) have unique pulmonary pathology and are highly susceptible to *M.tb* challenge, making them well-utilized for vaccine and drug therapy studies (40–42) and immune cell influx evaluation to areas of infection (43). Using these three differentially susceptible mouse strains we executed a head-to-head evaluation of i.n. BCG vaccination on bacterial burden and survival after aerosol infection with a lab-adapted and clinical *M.tb* strains.

## Results

Cohorts of C57BL/6, SWR, and C3HeB/FeJ mice were either vaccinated with 5 × 10^5^ CFU BCG intranasally or left untreated and 12 weeks later challenged with a low dose aerosol (LDA) using either laboratory *M.tb* H37Rv strain or clinical and highly virulent *M.tb* HN878 strain. Regardless of mouse strain or *M.tb* challenge strain, aerosol delivery of *M.tb* resulted in 20–67 CFU per mouse at 24 h (Table 1), in line with conventional LDA parameters.

**Table 1:** Bacterial counts at 24 hours post *M.tb* infection.

**Mouse strain**	**24 h CFU average**	
	***M.tb* H37Rv**	***M.tb* HN878**
C57BL/6	23.5	20.3
SWR	40.3	66.7
C3HeB/FeJ	30.3	41.4

N = 3 mice per group used for average counts.

**Figure 1:**
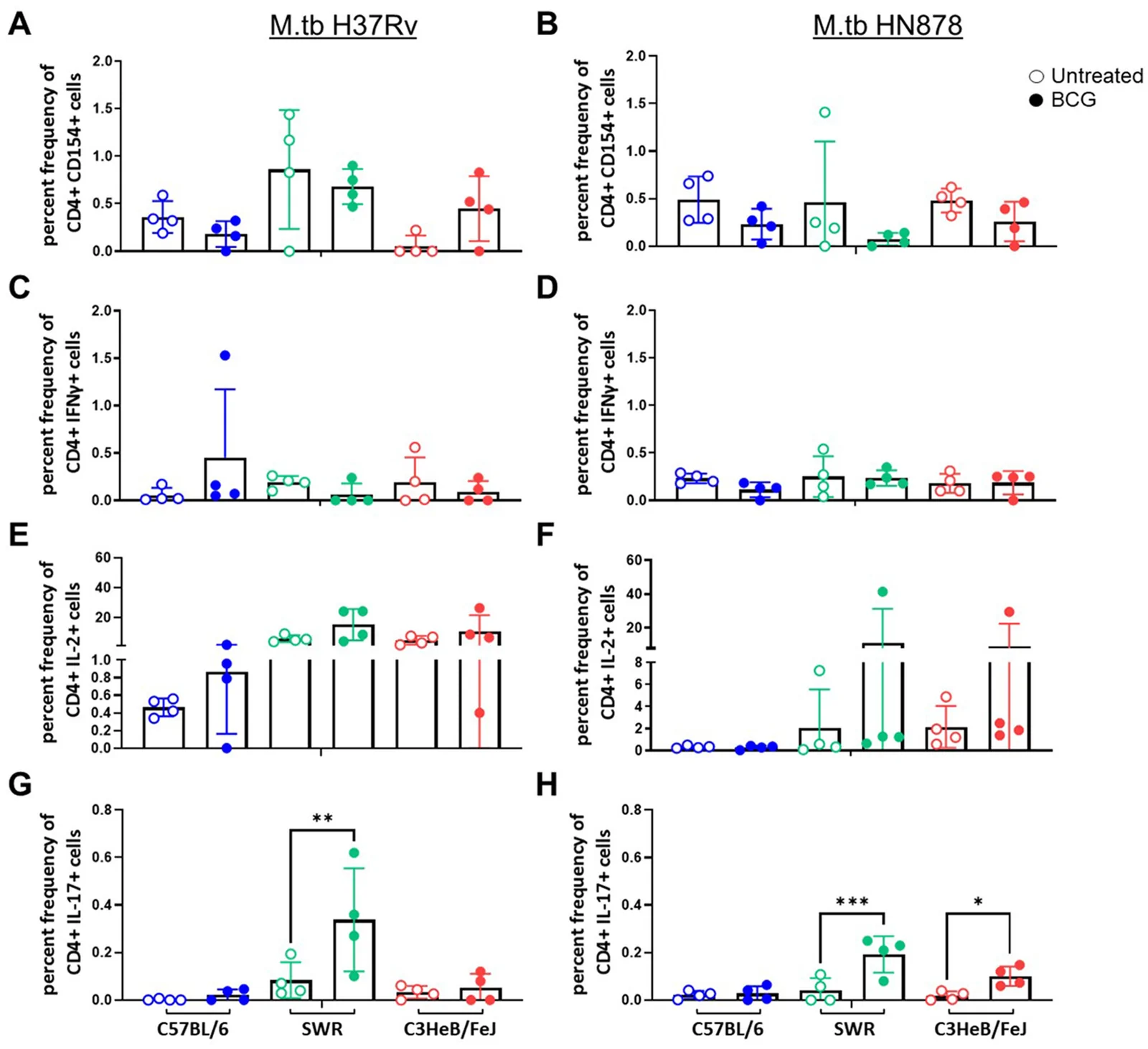
CD44+ CD4+ T cell proinflammatory cytokine expression after *ex vivo M.tb* H37Rv lysate stimulation. Percent frequency of CD44+ CD4+ cytokine expressing cells post stimulation with 10 μg/mL of *M.tb* H37Rv lysate from lung homogenates of *n* = 4 mice per group from either **(A, C, E)** *M.tb* H37Rv or **(B, D, F)** *M.tb* HN878 challenged cohorts 4 weeks post-infection. Untreated (open circles) cohorts were compared with i.n. BCG vaccinated (closed circles) cohorts of C57BL/6 (blue) SWR (green) or C3HeB/FeJ (red) mice. Expression of **(A, B)** CD154, **(C, D)** IFNγ, **(E, F)** IL-2, and **(G, H)** IL-17 were evaluated. One-Way ANOVA with multiple corrections for paired tests within mouse strain and between vaccination regimens used Šídák's multiple comparisons test. Significant differences noted in the figure where *p < 0.05, **p < 0.01, and ***p < 0.001.

At the 4-week post-infection timepoint we assessed cellular responses to *M.tb* H37Rv lysate (Figure 1). Antigen experienced (CD44+) CD4+ and CD8+ T cells were evaluated for CD154, CD107a, IL-2, IL-17, IL-21, IFNγ, and TNFα expression (gating strategy is presented in Supplementary Figure 1). We observed no significant differences between mouse strain or vaccination regimen in the cases of CD4+ T cells expressing IL-21, or TNFα after *ex vivo* stimulation with *M.tb* lysate (data not shown). Interestingly, by 4-weeks post-infection CD4+ T cells from SWR mice vaccinated with BCG demonstrated a significantly higher IL-17 response than unvaccinated controls (Figures 1G, H) and this pattern was similarly observed in the case of C3HeB/FeJ mice infected with *M.tb* HN878 (Figure 1H). When polyfunctional CD4+ T cells were evaluated for expression of two or more TH1 cytokines (IFNγ, IL-2, or TNFα), no statistically significant increases in BCG vaccinated cohorts were observed (Supplementary Figures 2A, B). However, in the case of mice infected with *M.tb* HN878, there was an increase in SWR BCG vaccinated mice with more CD4+ T cells co-expressing IL-17 and IL-2, and in BCG vaccinated C3HeB/FeJ mice having more CD4+ T cells co-expressing IL-17 with TNFα than matched unvaccinated cohorts (Supplementary Figures 3A, B). Otherwise, there are no notable trends of proinflammatory cytokine expression from CD4+ T cells across cohorts post-infection (Figure 1).

**Figure 2:**
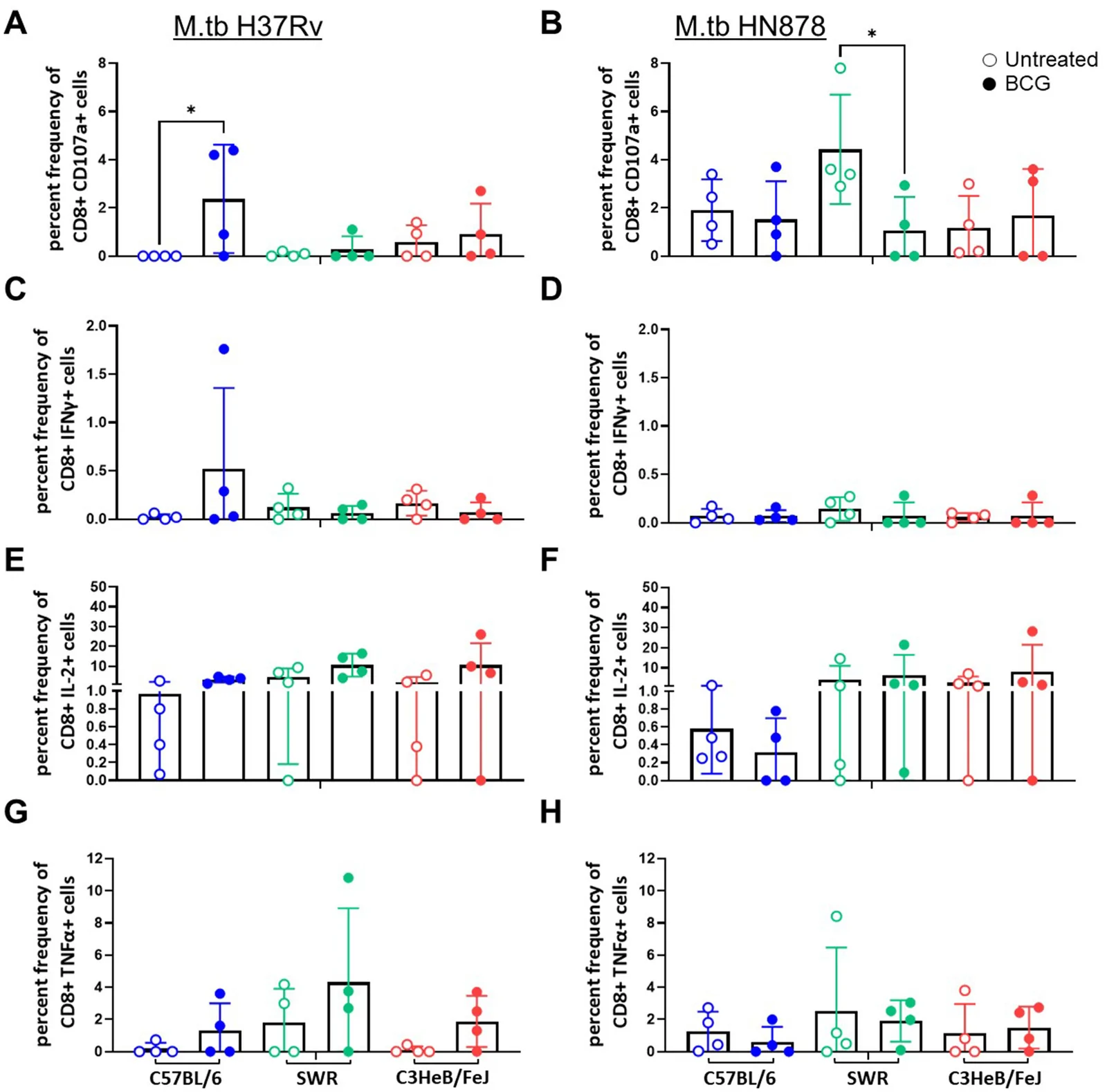
CD44+ CD8+ T cell proinflammatory cytokine expression after *ex vivo M.tb* H37Rv lysate stimulation. Percent frequency of CD8+ cytokine expressing cells post stimulation with 10 μg/mL of *M.tb* lysate from lung homogenates of *n* = 4 mice per group from either **(A, C, E)** *M.tb* H37Rv or **(B, D, F)** *M.tb* HN878 challenged cohorts 4 weeks post-infection. Untreated (open circles) cohorts were compared with i.n. BCG vaccinated (closed circles) cohorts of C57BL/6 (blue) SWR (green) or C3HeB/FeJ (red) mice. Expression of **(A, B)** CD107a, **(C, D)** IFNγ, **(E, F)** IL-2, and **(G, H)** TNFα were evaluated. One-Way ANOVA with multiple corrections for paired tests within mouse strain and between vaccination regimens used Šídák's multiple comparisons test. Significant differences noted in the figure where *p < 0.05.

We next evaluated CD8+ T cell cytotoxic activity and cytokine expression after *ex vivo M.tb* lysate stimulation by flow cytometry. We used CD107a as a marker of CD8+ T cell degranulation. Only in the case of C57BL/6 mice challenged with *M.tb* H37Rv did we observe a significant increase in CD107a staining in the BCG vaccinated group (Figure 2A), no significant differences were observed in *M.tb* HN878 challenged cohorts (Figure 2B). In the case of CD8+ T cells expressing proinflammatory cytokines we observed no major trends for IFNγ, where all cohorts had extremely low percent frequencies which did not change with *M.tb* strain or mouse strain (Figures 2C, D). Although not significant, there was a trend of higher CD8+ T cell IL-2 and TNFα expression in each mouse strain given i.n. BCG vaccination compared to untreated controls, 4-weeks after *M.tb* H37Rv challenge (Figures 2E, G). No similar trends were observed post-infection with *M.tb* HN878 for CD8+ T cell cytokines (Figures 2F, H).

In addition to total responses after *ex vivo* stimulation with *M.tb* lysate, antigen specific responses were evaluated. This included antigen Rv3874 (CFP-10) which is only expressed by *M.tb* as it is located in a region of deletion within BCG. At 4-weeks post-infection lung homogenate samples were assessed as described above for both CD4+ and CD8+ T cell cytokine expression. For CD4+ T cells, SWR mice with BCG vaccination consistently expressed significantly higher IL-2, IL-17 (Supplementary Figure 4) IL-21, and TNFα (data not shown) cytokines *ex vivo* compared with unvaccinated SWR controls following challenge with *M.tb* H37Rv, but this pattern was not observed in immunized SWR mice challenged with *M.tb* HN878. We observed BCG vaccinated SWR mice had a statistically higher percent frequency of CD4+ T cells co-expressing IL-2 and TNFα than unvaccinated SWR mice challenged with *M.tb* H37Rv (Supplementary Figure 2C). In addition, BCG vaccinated SWR cohorts demonstrated significantly higher CD4+ T cells co-expressing IL-17 with IL-2 than matched unvaccinated cohorts (Supplementary Figure 3C). C57BL/6 and C3HeB/FeJ mice were consistently low responders in each condition and no significant differences were observed between unvaccinated vs. BCG vaccinated comparisons for single or polyfunctional TH1 CD4+ T cells. Cohorts vaccinated with i.n. BCG and challenged with *M.tb* H37Rv generally trended higher in CD8+ T cell responses with *ex vivo* Rv3874 antigen stimulation. CD8+ T cells from SWR mice prophylactically immunized with i.n. BCG expressed significantly more IFNγ and IL-2 after *ex vivo* Rv3874 stimulation compared with unvaccinated controls (Supplementary Figure 5).

**Figure 3:**
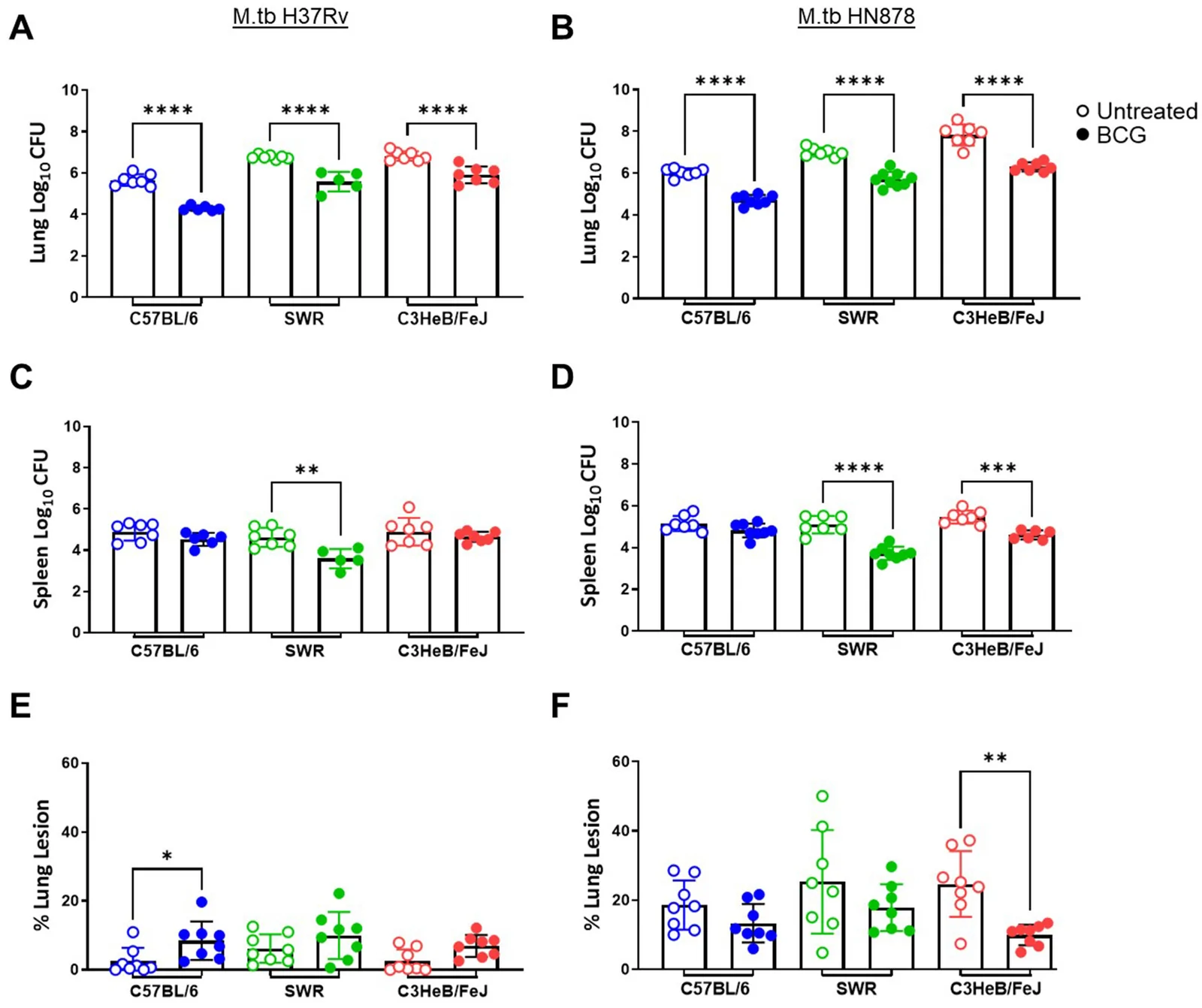
Pulmonary and disseminated bacterial burden and pathology 4 weeks post-infection. **(A, B)** Lung and **(C, D)** Spleen homogenate Log_10_ CFU counts and **(E, F)** percent lung lesion area from H&E stained accessory lobe slides evaluated 4 weeks post-infection with a LDA of **(A, C, E)** *M.tb* H37Rv or **(B, D, F)** *M.tb* HN878. Untreated groups are open circles and BCG vaccinated groups are noted as closed circles. One-Way ANOVA with multiple corrections for paired tests within mouse strain and between vaccination regimens used Šídák's multiple comparisons test. Significant differences noted in the figure where *p < 0.05, **p < 0.01, ***p < 0.001, and ****p < 0.0001. *n* = 5–7 per group.

At 16-weeks post-vaccination and 4-weeks post-infection, bacterial burden was evaluated in each cohort. Lung Log_10_ CFU as well as spleen Log_10_ CFU were enumerated to evaluate the primary site of infection and representative organ for dissemination, respectively. Across each mouse and *M.tb* strain, prior i.n. vaccination with BCG resulted in significant protection in the lungs compared to untreated controls (Figures 3A, B). As expected, untreated susceptible mouse strains SWR and C3HeB/FeJ harbored significantly higher pulmonary bacterial burden than relatively resistant C57BL/6 in both infection schemes. Interestingly, unvaccinated C3HeB/FeJ mice had significantly higher lung bacterial burden than unvaccinated SWR mice at 4-weeks post-infection with *M.tb* HN878 but not *M.tb* H37Rv (Tables 2, 3). In the spleen, SWR mice with BCG vaccination were protected from both *M.tb* H37Rv and *M.tb* HN878 dissemination compared to unvaccinated controls, and C3HeB/FeJ mice showed significantly reduced bacterial burden compared to unvaccinated controls in the case of virulent *M.tb* HN878 infection (Figures 3C, D and Table 3). Unvaccinated cohorts infected with *M.tb* HN878 contained more heterogeneity and higher total percent lung lesion area, measured from accessory lobes stained with H&E, than unvaccinated controls infected with *M.tb* H37Rv by this 4-week post-infection timepoint (Figures 3E, F). There was a consistent trend of less lung lesion area induced by *M.tb* HN878 infection in BCG vaccinated groups, but this was only significantly lower in C3HeB/FeJ cohorts (Figure 3E). While C57BL/6 mice received the relatively lowest infection at the time of infection (Table 1), this cohort experienced nearly identical dissemination to the spleen across each *M.tb* challenge by 4-weeks post-infection (Table 3), suggesting the lack of protection in the BCG vaccinated animals from *M.tb* dissemination in this mouse strain was not due to initial *M.tb* deposition.

**Table 2:** Comparison of bacterial burden in unvaccinated mouse strains.

**Mouse strain**	***M.tb*** **H37Rv**		***M.tb*** **HN878**	
	**Lung**	**Spleen**	**Lung**	**Spleen**
C57BL/6 x SWR	<0.0001^****^	0.6014 (n.s.)	0.0001^***^	0.9592 (n.s.)
C57BL/6 x C3HeB/FeJ	<0.0001^****^	0.9998 (n.s.)	<0.0001^****^	0.2800 (n.s.)
SWR x C3HeB/FeJ	0.8986 (n.s.)	0.6436 (n.s.)	0.0007^***^	0.1809 (n.s.)

Log_10_ CFU compared between cohorts using ordinary One-Way ANOVA with Tukey's multiple comparisons test, adjusted p-values reported.

n.s., not significant. ^***^p < 0.001 and ^****^p < 0.0001 denote degrees of significant differences by One-Way ANOVA.

**Table 3:** Bacterial burden 4 weeks post-infection.

**Mouse strain**	**Vaccination**	**Log**_**10**_ **CFU** ±**SD** ***M.tb*** **H37Rv**		**Log**_**10**_ **CFU** ±**SD** ***M.tb*** **HN878**	
		**Lung**	**Spleen**	**Lung**	**Spleen**
C57BL/6	Untreated	5.66 ± 0.28	4.90 ± 0.43	6.03 ± 0.20	5.15 ± 0.37
	BCG	4.30 ± 0.12	4.53 ± 0.31	4.72 ± 0.24	4.82 ± 0.33
SWR	Untreated	6.78 ± 0.10	4.63 ± 0.46	7.01 ± 0.21	5.10 ± 0.42
	BCG	5.58 ±.047	3.59 ± 0.47	5.71 ± 0.35	3.72 ± 0.32
C3HeB/FeJ	Untreated	6.83 ± 0.23	4.90 ± 0.67	7.83 ±.051	5.47 ± 0.31
	BCG	5.91 ± 0.41	4.65 ± 0.24	6.30 ± 0.21	4.61 ± 0.21

Mean Log_10_ CFU from n = 5–7 per group.

**Figure 4:**
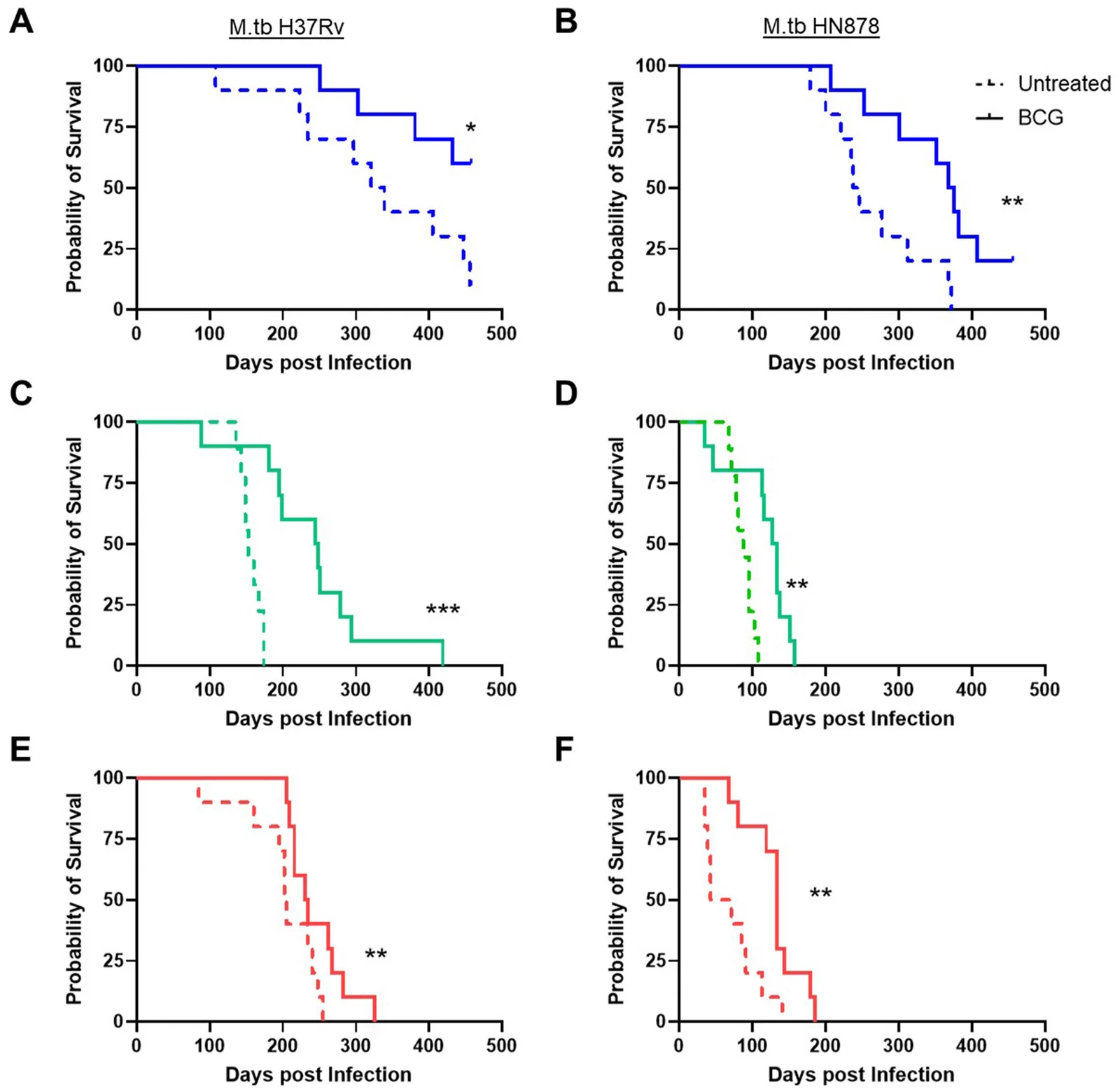
Survival of challenged cohorts and durable protection from mucosal BCG vaccination. Probability of survival, where *n* = 10 animals per group were weighed weekly and assessed for morbidity criteria post-infection with either **(A, C, E)** *M.tb* H37Rv or **(B, D, F)** *M.tb* HN878. Untreated (dashed line) cohorts were compared with i.n. BCG vaccinated (solid line) cohorts of **(A, B)** C57BL/6 (blue) **(C, D)** SWR (green) or **(E, F)** C3HeB/FeJ (red) mice. Comparisons with or without BCG vaccination within a mouse and *M.tb* challenge used a Log-rank (Mantel-Cox) test, significant differences are labeled accordingly *p < 0.05, **p < 0.01, and ***p < 0.001.

Lastly, durability of vaccine-mediated protection from morbidity was evaluated in *n* = 10 animals per group which were weighed weekly post-infection. Animals were observed for moribund characteristics or behaviors and met objective euthanasia criteria when weight loss exceeded 20% of maximum. We compared virulence of *M.tb* strains by median survival days across challenges as well as Log-rank hazard ratio evaluation of the full survival curves. For unvaccinated SWR and C3HeB/FeJ mice challenged with *M.tb* HN878 the median survival days was roughly half or worse of that for the same untreated cohorts challenged with *M.tb* H37Rv (Table 4). Overall, we observed that *M.tb* HN878 infection was more virulent over time by Log-rank Mantel-Cox test in unvaccinated SWR (*p* < 0.0001) and C3HeB/FeJ (*p* = 0.0001) mouse backgrounds compared to similarly challenged unvaccinated *M.tb* H37Rv cohorts, but not in unvaccinated C57BL/6 cohorts by the same test (*p* = 0.1477). In each mouse and *M.tb* challenge combination, i.n. BCG vaccination afforded greater cohort median survival days compared to untreated controls (Table 4) and provided durable protection from morbidity by probability of survival log-rank tests in each *M.tb* infection scheme within each mouse strain tested (Figure 4).

**Table 4:** Median survival days with and without mucosal BCG vaccination.

**Mouse strain**	***M.tb*** **H37Rv median survival (d)**		**Log-rank *p* between *M.tb* H37Rv curves**	***M.tb*** **HN878 median survival (d)**		**Log-rank *p* between *M.tb* HN878 curves**
	**Untreated**	**BCG**		**Untreated**	**BCG**	
C57BL/6	330	> 458	0.236^*^	242	>368	0.0070^**^
SWR	153	246	0.0002^***^	88	130	0.0022^**^
C3HeB/FeJ	202	231	0.0035^**^	57	134	0.0032^**^

Log-rank (Mantel-Cox) test. Study terminated at 458 days post-infection. Within challenge cohort differences were evaluated with a Log-rank test where ^*^p < 0.05, ^**^p < 0.01, and ^***^p < 0.001.

## Conclusions/discussion

Here we demonstrate that mucosally delivered BCG affords significant protection from pulmonary bacterial burden and enhances survival in all mouse background and *M.tb* challenge settings evaluated in this study. However, protection from dissemination was variable as was the rate of decline with respect to survival outcomes. In the case of both SWR and C3HeB/FeJ mice challenged with *M.tb* HN878 there is a stark and rapid decline of the group, possibly suggesting a more uniform sensitivity than other combinations. These data serve as a reference for those needing survival as an endpoint, who may need to execute studies in shorter windows of time (SWR or C3HeB/FeJ x *M.tb* HN878) or examine the natural or vaccine-induced heterogeneity of an infection (C57BL/6 x *M.tb* H37Rv). Furthermore, we observed a significant reduction in percent lung lesion area in BCG-vaccinated C3HeB/FeJ mice compared to matched unvaccinated cohorts by 4-weeks post-infection with *M.tb* HN878 which is a fairly early time point for pathology assessments. These data are important given the power of translational pathological outcomes in C3HeB/FeJ mice.

Additionally, there is a significant and expected contribution of *M.tb* challenge strain on outcomes, where highly virulent *M.tb* HN878 infection resulted in higher bacterial burden, more immunopathology by 4-weeks post-infection and reduced survival and yet did not result in blatantly higher CD4+ or CD8+ T cell responses with *ex vivo* stimulation. While surprising, this is in line with prior observations where infection with *M.tb* Beijing strains, including *M.tb* HN878, over time induced an expansion of regulatory FoxP3+ T cells, downregulating effector-led immunity driven by BCG vaccination (44). The relatively weak and sometimes reduced expression of CD107a on CD8+ T cells derived from lung samples after *ex vivo* stimulation with *M.tb* lysate or recombinant antigen Rv3874 is congruent with human clinical studies which also observed reduced CD107a staining from lymph node samples compared to peripheral blood samples obtained from tuberculous lymphadenitis patients (45). Together these data may indicate a *M.tb* induced regulatory profile that is more acute closer to the site of infection.

Interestingly, BCG vaccinated SWR mice demonstrated a consistent increase in CD4+ T cells expressing IL-2 or IL-17 or both after *ex vivo* stimulation, which is notably lacking from the other two mouse strains. It is of interest in follow on studies to evaluate whether this contribution from IL-2 and IL-17 also contribute or correlate with reduced systemic and disseminated bacterial burden which was also observed in the SWR cohorts that received BCG vaccination. Aguilo et al. showed IL-17 dependent protection following pulmonary delivery of BCG, measured by a reduction in lung and spleen bacterial burden, and survival against *M.tb* H37Rv in the *M.tb* susceptible DBA/2 mouse strain (24). Interestingly, in this same study, BCG given by the i.d. route did not result in protection in the susceptible DBA/2 mice, but did protect wildtype C57BL/6 mice (24). Our group has also shown that mucosal delivery with a subunit vaccine is capable of inducing a skewed T helper 17 (TH17) immune response (46). Although we saw robust pulmonary *M.tb* protection following mucosal BCG immunization regardless of mouse background or *M.tb* strain in our current study, less consistent protection against *M.tb* was observed in the spleen. A limitation of all TB vaccine studies is that the field has not delivered discrete immune correlates of protection, and indeed we hypothesize that it is more likely that a suite of responses (47) are working together to limit *M.tb in vivo* and that many immune pathways together can garner similar results. It is unclear from these studies whether the CD4+ or CD8+ T cell responses from 4-weeks post-infection across mouse strains are meaningful toward protection and survival responses mediated by i.n. BCG vaccination or other immune parameters are driving those responses, such as T resident memory cells, humoral immune responses or specific trained innate immunity in the airway. The studies presented here are limited in that flow panels did not include markers that allow the discrimination of lung tissue-resident CD4+ and CD8+ T cells from those in the lung vasculature, which may further resolve differences in protection mediated by BCG vaccination. It has been observed that monocyte recruitment to the lungs after intradermal (i.d.) BCG vaccination correlated with protection in C57BL/6 mice, rather than T cell recruitment (48). These data together with CC models suggest that different host genetics in combination with BCG vaccination may pave different paths toward immune protection. All of these endpoints are worth pursuing.

Historically, oral BCG and i.d. BCG were evaluated for use in humans and ultimately i.d. was adopted. However, the oral route generates high-quality mucosal responses which has been observed in both mice and humans (49–51). Studying the unconventional administration of BCG (25) is making a resurgence in the TB vaccine community as a means to help define immune correlates of protection. This includes the notable use of intravenous (i.v.) delivery in non-human primate models that resulted in protection and robust immune endpoints across cohorts (52–55). In a diversity outbred mouse model, i.v. BCG enhanced survival but did not significantly reduce pulmonary *M.tb* burden compared to cohorts vaccinated via the i.d. route (56). Indeed, this may be due in part to the breadth of antigen-specific responses in i.v. vaccinated cohorts being larger than traditionally immunized i.d. mouse cohorts (57). While i.d. delivered BCG is controlled and cleared from the host overtime [in as little 12 weeks in mice as reported here (29)], it is unclear whether mucosal i.n. or systemic i.v. delivery of the vaccine would present novel niches for more long term persistence. This is part of ongoing discussions and preclinical studies about the safety of administering BCG i.v. clinically (25, 58). In addition to route, vaccine dose is known to be critical in meeting specific thresholds of protection against *M.tb* challenge in both mice (59) and other preclinical models (25, 53). Further work could compare routes and doses in mice, including i.n. and i.v. with traditional s.c./i.d. BCG, to obtain meaningful correlates that drive protection in the mouse model of TB. Cumulatively, these data suggest there is still much we can glean from BCG vaccination strategies if we vary the route or dose of delivery and carefully interrogate the interplay with different host or pathogen genetics.

## Materials and methods

### Mouse vaccination and challenge

Female C57BL/6, SWR, and C3HeB/FeJ mice were purchased from Charles River Laboratories at 5–8-weeks old. Mice were housed at (renamed post-receivership) the Infectious Disease Research Institutes (IDRI) biosafety level 3 (BSL-3) animal facility under pathogen-free conditions and were handled in accordance with approved protocols from the IDRI Institutional Animal Care and Use Committee (IACUC). The institute, formerly known as IDRI, operated under USDA Certificate # 91-R-0061 and PHS Assurance # A4337-01. All methods were carried out in accordance with animal welfare guidelines and regulations. For vaccination, mice were given an intranasal immunization of 5 × 10^5^ colony forming units (CFU) of BCG Aeras Pasteur [as previously reported in (19)] in a total of 50 μl, 25 μl per nare, while under ketamine/xylazine anesthesia. The sequenced genome of BCG Aeras Pasteur propagated by our lab and maintained as vaccination stocks can be found at GenBank ID: SAMN38797945. Twelve weeks later, mice were challenged with a low dose aerosol (LDA, 25-100 CFU per mouse upon infection) of either *M.tb* H37Rv or *M.tb* HN878 (BEI Resources) using a Madison aerosol infection chamber. Delivery and deposition of *M.tb* dose was confirmed 24 h post-infection by evaluating bacterial burden from the lungs as described below. Ten mice per group were followed after *M.tb* infection for survival analysis. Animals with >20% weight loss, or moribund condition, were euthanized. The survival study was prematurely ended with C57BL/6 animals still not meeting endpoint criteria on day 458 post-infection, due to the Authors' prior institution (IDRI) abruptly entering receivership.

### Intracellular cytokine staining and flow cytometry

Intracellular staining and flow cytometry was performed on lung homogenates 4 weeks post-*M.tb* infection with either *M.tb* H37Rv or *M.tb* HN878. Samples were incubated in red blood cell lysis buffer (eBioscience), washed and resuspended in RPMI 1640 (Life Technologies) + 10% fetal bovine serum (FBS; BioWhittaker, Inc.) and subsequently aliquoted in 96-well round bottom plates. Cells were then stimulated with media alone, 10 μg/mL *M.tb* H37Rv whole cell lysate, 10 μg/mL of recombinant *M.tb* antigens Rv3478 (PPE60 Mtb39c), Rv3874 (CFP10), Rv3875 (ESTAT6), or 1 μg/mL phorbol myristate acetate (PMA; Calbiochem) + 1 μg/mL ionomycin (Sigma) and incubated at 37°C. During the stimulation fluorochrome-conjugated anti-mouse CD107a antibody (LAMP-1, clone 1D4B, BioLegend) was also added to the plates. After 1–2 h 1 μg/μl of GolgiPlug (BD Biosciences) was added and samples were incubated at 37°C for an additional 8 h. Samples then remained at 4°C until staining (<12 h). Samples were first surface stained with fluorochrome-conjugated antibodies against mouse CD4 (clone RM4-5, BioLegend), CD8 (clone 53-6.7, BioLegend), and 1 μg/mL of Fc receptor block anti-CD16/CD32 (clone 93, eBioscience) in PBS with 1% bovine serum albumin (BSA) for 10–15 min at room temperature (RT). Cells were then washed and fixed using BD Biosciences Fix/Perm reagent for 20 min at RT. Subsequently samples were washed with BD Perm/Wash followed by intracellular staining in Perm/Wash reagent with anti-mouse IFN-γ (clone XMG1.2, Invitrogen), IL-2 (clone JES6-5H4, eBioscience), IL-17A (clone Tc11-1BH10.1, BioLegend), TNF-α (clone MP6-XT22, eBioscience), CD44 (clone IM7, BioLegend), IL-21 (clone mhalx21, ThermoFisher Scientific), and CD154 (clone MR7, eBioscience) for 10 min at RT. All antibodies were used at 10 μl/mL. The samples were then incubated in 4% paraformaldehyde for 20 min to fix, decontaminate, and remove from the containment facility before washing and resuspension in PBS + 1% BSA. An LSRFortessa flow cytometer (BD Bioscience) was used for sample acquisition, and analysis was performed using FlowJo v10 (BD Biosciences).

### Cross section evaluation of bacterial burden

To enumerate bacterial burden in infected animals, organs were homogenized at 24 h (*n* = 3, lung only) or 4 weeks (*n* = 5–8, lung and spleen) post-infection as previously described (60, 61). Lung and spleen tissue from infected animals was strain and homogenized in 2–5 mL of either RPMI + FBS (lung) or PBS + Tween-80 (Sigma-Aldrich) CFU buffer (spleen) using an Omni tissue homogenizer (Omni International, Kennesaw, GA). Homogenized tissue was plated neat or across a series of dilutions on Middlebrook 7H10 agar plates (Fisher Scientific, Waltham, MA) and subsequently incubated at 37°C and 5% CO_2_ for ~ 3 weeks before colonies were counted. For CFU plating, 5 μg/ml of thiophene-2-carboxylic acid hydrazide (TCH) was supplemented to 7H10 agar to inhibit the growth of BCG and to allow the growth of *M.tb* in duplicate plates. Bacterial burden as CFU/mL was calculated per organ and is presented here as Log_10_ values. Reduction in bacterial burden was calculated as the difference in mean Log_10_ values between groups assessed.

### Histopathology

Lung accessory lobes from 4 weeks post *M.tb* infection were collected and temporarily stored in 10% normal buffered formalin. Hematoxylin and eosin (H&E) slides were generated at the Benaroya Research Institute Histology Core facility (Seattle, WA, USA). Blinded slides were analyzed by veterinary pathologist (Dr. Brendan Podell, Colorado State University). Image analysis and lesion area identification and percent lesion area was performed as previously described (35, 61).

### Statistical analysis

For 4-week CFU, percent lesion area and ICS data comparing groups with or without BCG vaccination within a mouse and *M.tb* challenge, a One-Way ANOVA with multiple corrections for paired tests within mouse strain using Šídák's multiple comparisons test was used. The flow cytometry data were assessed using FlowJo v10 (BD), and SPICE (NIH, https://niaid.github.io/spice/). For survival comparisons with or without BCG vaccination within a mouse and *M.tb* challenge a Long-rank (Mantel-Cox) test was used and median days of survival for each group are reported in the text. Statistical analyses were performed using GraphPad Prism 10.2.2 software (GraphPad Software, San Diego, CA). Grubbs tests were run to identify potential outliers within cohorts. Significant differences are labeled accordingly in the figures where ^*^p < 0.05, ^**^p < 0.01, ^***^p < 0.001, and ^****^p < 0.0001.

## Data Availability

The original contributions presented in the study are included in the article/Supplementary material, further inquiries can be directed to the corresponding author.
