# Supplementary material for: Mucosal BCG delivery provides a spectrum of protection from different Mycobacterium tuberculosis strains across susceptible and resistant mouse backgrounds
Source: Front Tuberc. 2024 Oct 15;2:1417939. doi: 10.3389/ftubr.2024.1417939 (PMC13494978; doi:10.3389/ftubr.2024.1417939)
Supplement: Supplementary file 1 [file Data_Sheet_1.pdf]

Supplementary Material

Mucosal BCG delivery provides a spectrum of protection from different *Mycobacterium tuberculosis* strains across susceptible and resistant mouse backgrounds

Sasha E. Larsen[1], Brittany D. Williams[1,2], Tiffany Pecor[1], Valerie A. Reese[1], Zhiyi Zhu[1], Dana E. Miller[1], Brendan K. Podell[3], Susan L. Baldwin[1], Rhea N. Coler[1,2,4]

[1]Seattle Children's Research Institute, Center for Global Infectious Disease Research, Seattle Children's, Seattle, WA, USA

[2]University of Washington, Department of Global Health, Seattle, WA, USA.

[3]Department of Microbiology, Immunology and Pathology, Colorado State University, Fort Collins, CO, USA

[3]University of Washington School of Medicine, Department of Pediatrics, Seattle, WA, USA.

*** Correspondence:**
Rhea N. Coler
Rhea.Coler@Seattlechildrens.org

1 Supplementary Figures

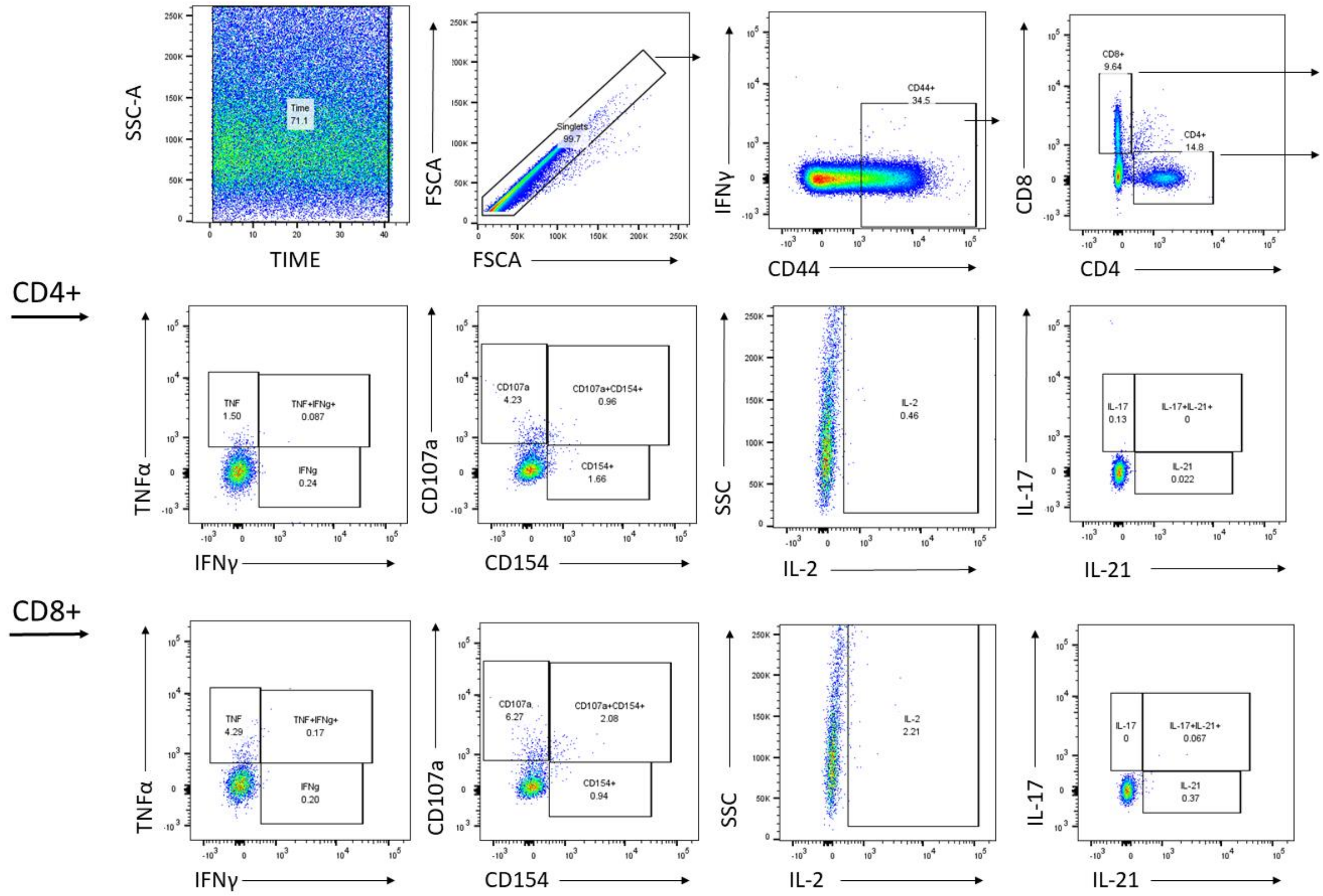

Supplementary Figure 1. Cells recovered from lung tissue homogenate 4-weeks post-infection were gated from singlets and antigen experienced CD44+ cells before being discriminated into CD4+ or CD8+ T cells. Subsequently CD4+ and CD8+ T cells were gated for expression of cytokines (TNFα, IFNγ, CD154, CD107a, IL-2, IL-21, and IL-17) in discrete subsets.

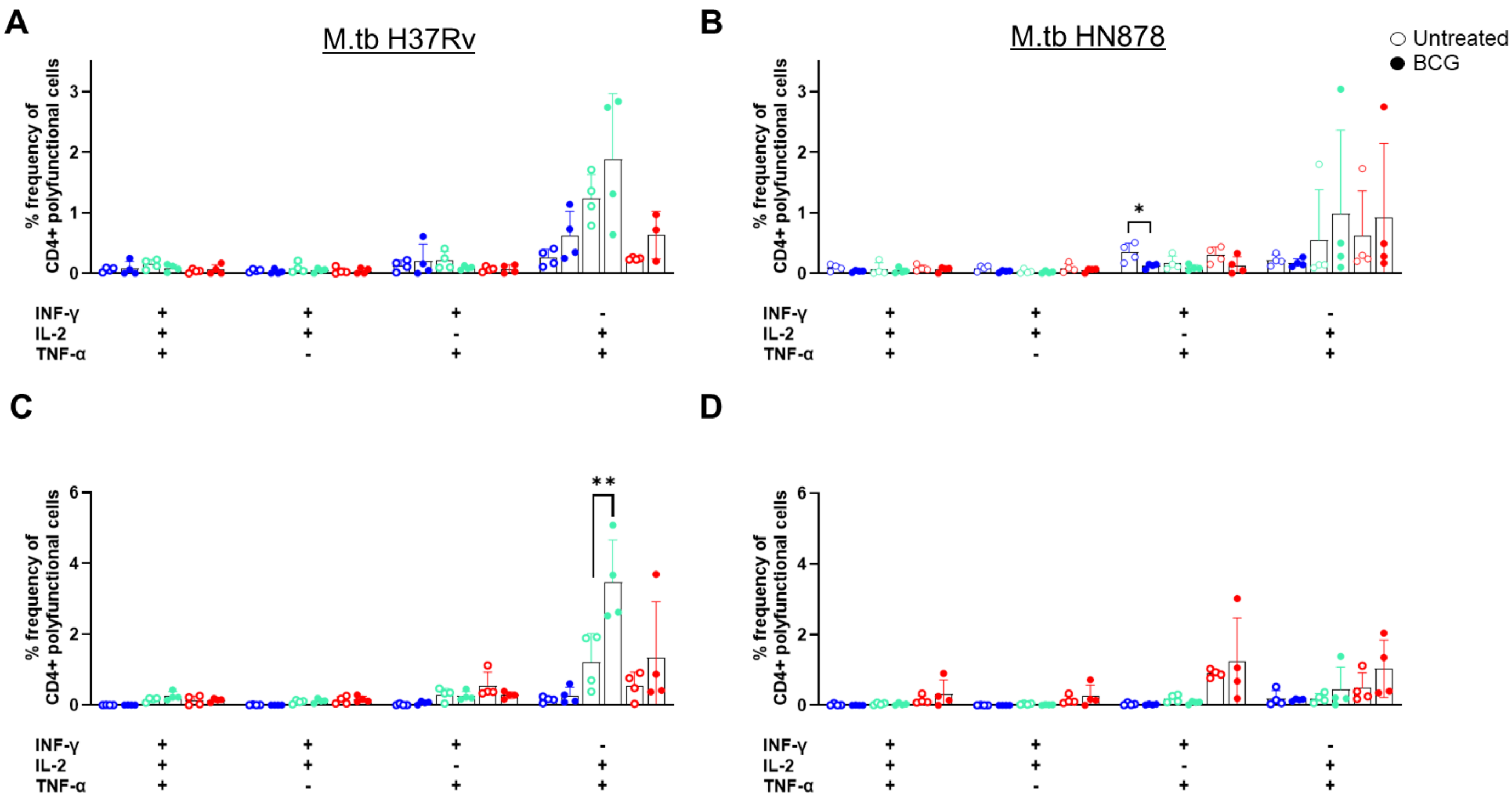

Supplementary Figure 2. Polyfunctional TH1 CD4+ T cell cytokine expression after *ex vivo* stimulation with **A, B**) *M.tb* lysate or **C, D**) Rv3874 (CFP-10). Percent frequency of CD4+ T cells expressing two or more of the following cytokines: IFNγ, IL-2 or TNFα are shown from lung homogenates of n=4 mice per group from either **A, C**) *M.tb* H37Rv or **B, D**) *M.tb* HN878 challenged cohorts four weeks post-infection. Untreated (open circles) cohorts were compared with i.n. BCG vaccinated (closed circles) cohorts of C57BL/6 (blue) SWR (green) or C3HeB/FeJ (red) mice. One-Way ANOVA with multiple corrections for paired tests within mouse strain and between vaccination regimens used Šídák's multiple comparisons test. Significant differences noted in the figure where * $p < 0.05$, and ** $p < 0.01$.

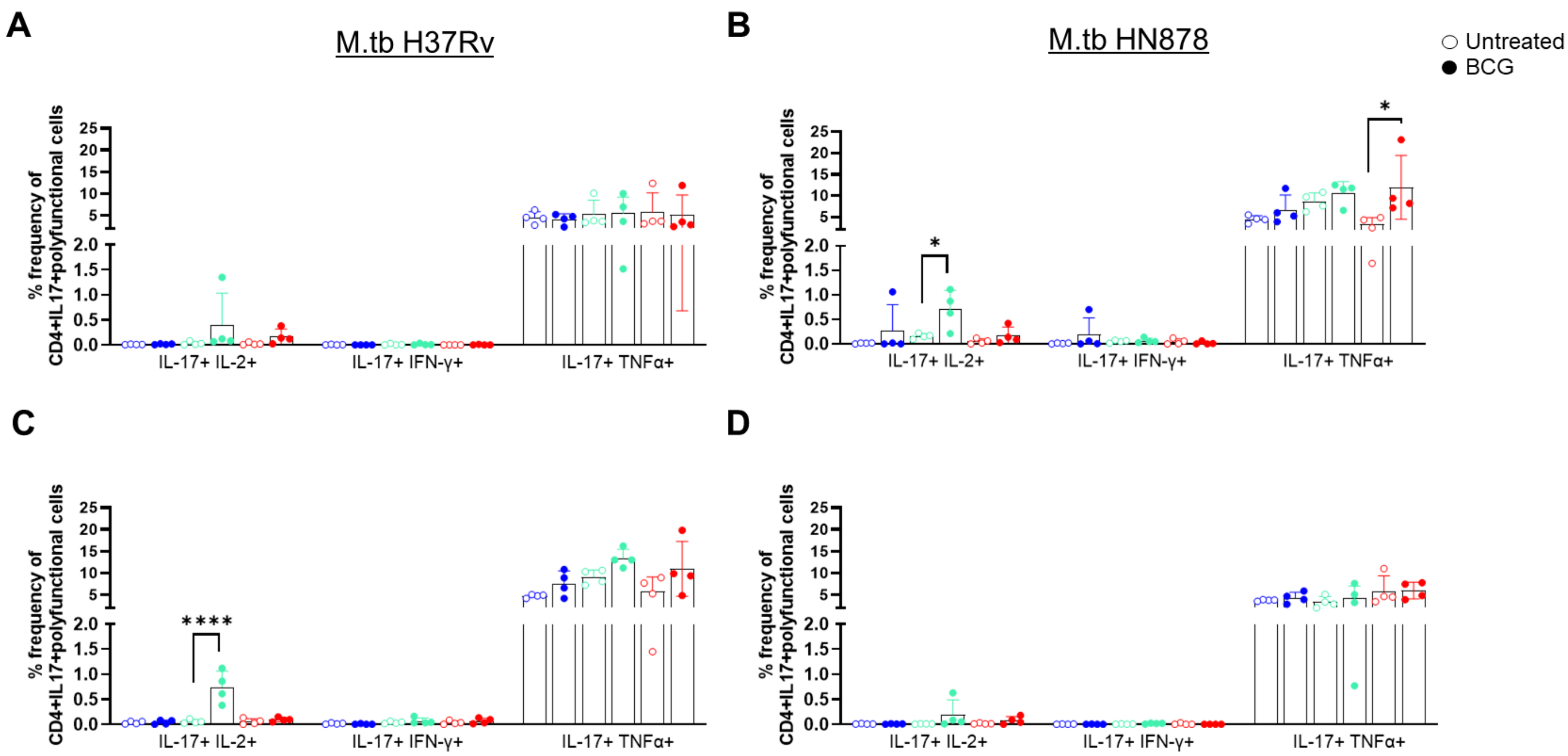

Supplementary Figure 3. Polyfunctional TH17 CD4+ T cell cytokine expression after *ex vivo* stimulation with **A, B**) *M.tb* lysate or **C, D**) Rv3874 (CFP-10). Percent frequency of CD4+ T cells expressing IL17 in addition to one or the following cytokines: IFNγ, IL-2 or TNFα are shown from lung homogenates of n=4 mice per group from either **A, C**) *M.tb* H37Rv or **B, D**) *M.tb* HN878 challenged cohorts four weeks post-infection. Untreated (open circles) cohorts were compared with i.n. BCG vaccinated (closed circles) cohorts of C57BL/6 (blue) SWR (green) or C3HeB/FeJ (red) mice. One-Way ANOVA with multiple corrections for paired tests within mouse strain and between vaccination regimens used Šídák's multiple comparisons test. Significant differences noted in the figure where * $p < 0.05$ and **** $p < 0.0001$.

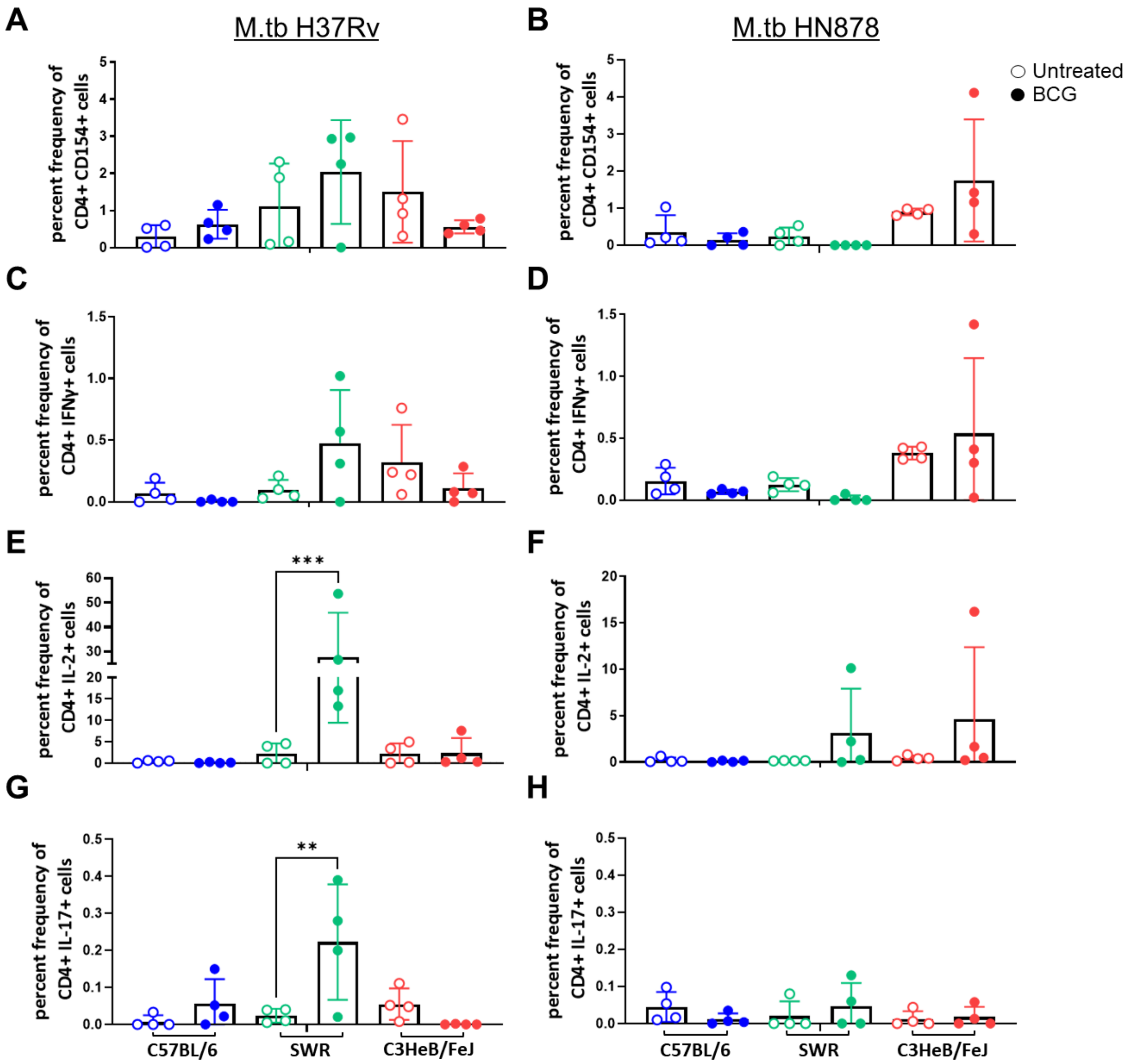

Supplementary Figure 4. CD44+ CD4+ T cell proinflammatory cytokine expression after *ex vivo* Rv3874 (CFP-10) stimulation. Percent frequency of CD44+ CD4+ cytokine expressing cells post stimulation with 10µg/mL of recombinant Rv3874 antigen isolated from lung homogenates of n=4 mice per group from either **A, C, E**) *M.tb* H37Rv or **B, D, F**) *M.tb* HN878 challenged cohorts four weeks post-infection. Untreated (open circles) cohorts were compared with i.n. BCG vaccinated (closed circles) cohorts of C57BL/6 (blue) SWR (green) or C3HeB/FeJ (red) mice. Expression of **A, B**) CD154, **C, D**) IFNγ, **E, F**) IL-2 and **G, H**) IL-17 were evaluated. One-Way ANOVA with multiple corrections for paired tests within mouse strain and between vaccination regimens used Šídák's multiple comparisons test. Significant differences noted in the figure where ** $p < 0.01$, and *** $p < 0.001$.

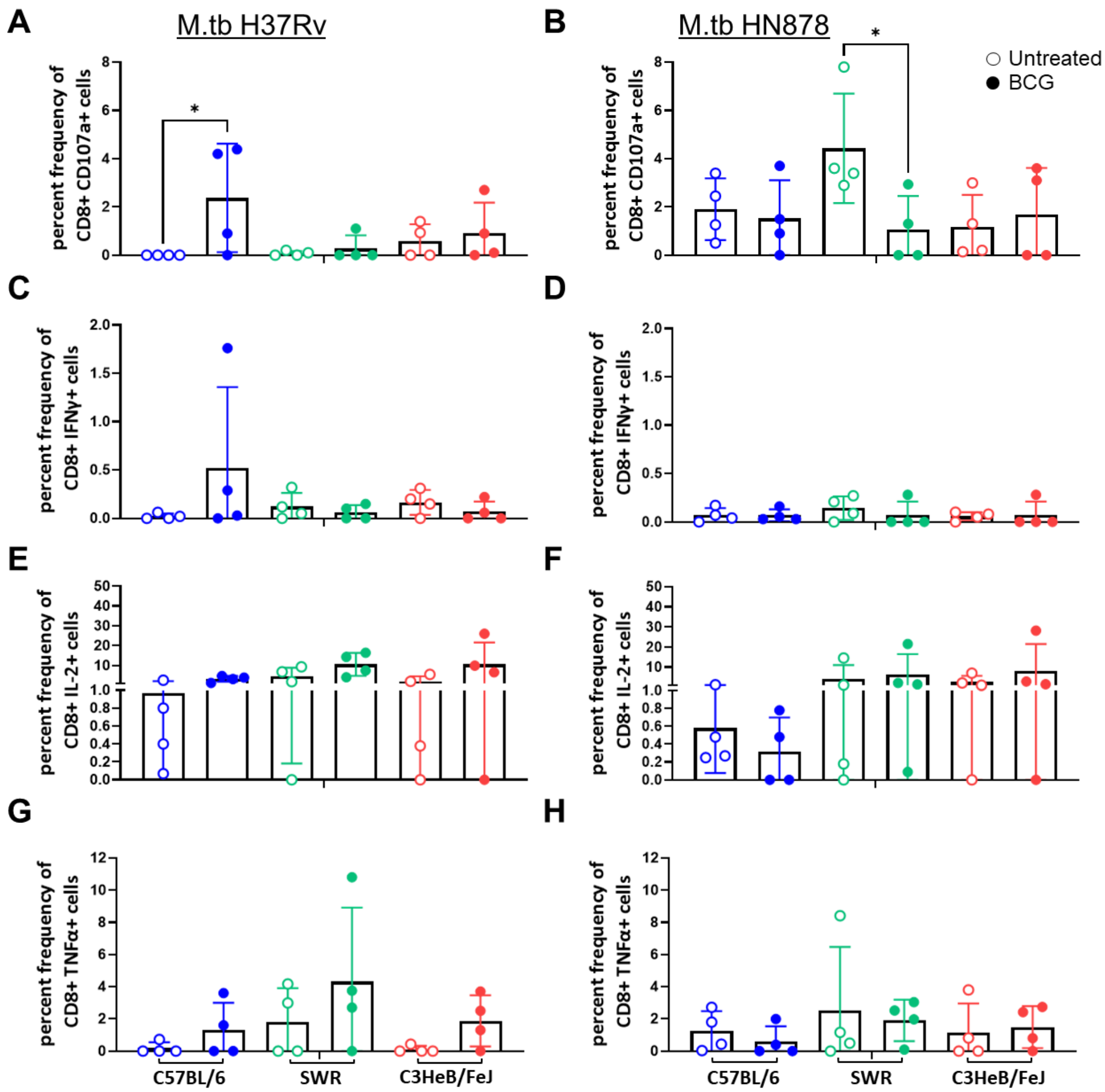

Supplementary Figure 5. CD44+ CD8+ T cell proinflammatory cytokine expression after *ex vivo* Rv3874 (CFP-10) stimulation. Percent frequency of CD44+ CD4+ cytokine expressing cells post stimulation with 10µg/mL of recombinant Rv3874 antigen isolated from lung homogenates of n=4 mice per group from either **A, C, E**) *M.tb* H37Rv or **B, D, F**) *M.tb* HN878 challenged cohorts four weeks post-infection. Untreated (open circles) cohorts were compared with i.n. BCG vaccinated (closed circles) cohorts of C57BL/6 (blue) SWR (green) or C3HeB/FeJ (red) mice. Expression of **A, B**) CD107a, **C, D**) IFNγ, **E, F**) IL-2 and **G, H**) TNFα were evaluated. One-Way ANOVA with multiple corrections for paired tests within mouse strain and between vaccination regimens used Šídák's multiple comparisons test. Significant differences noted in the figure where * $p < 0.05$.
